# Responses of Leaf Anatomy and CO_2_ Concentrating Mechanisms of the Aquatic Plant *Ottelia cordata* to Variable CO_2_

**DOI:** 10.3389/fpls.2020.01261

**Published:** 2020-08-14

**Authors:** Wenmin Huang, Shijuan Han, Zhenfei Xing, Wei Li

**Affiliations:** ^1^ Key Laboratory of Aquatic Botany and Watershed Ecology, Wuhan Botanical Garden, Center of Plant Ecology, Core Botanical Gardens, Chinese Academy of Sciences, Wuhan, China; ^2^ Aix Marseille Univ CNRS, BIP UMR 7281, IMM, FR 3479, Marseille, France; ^3^ University of Chinese Academy of Sciences, Beijing, China; ^4^ Institute of Hydrobiology, Chinese Academy of Sciences, Wuhan, China

**Keywords:** CO_2_ availability, C_4_, crassulacean acid metabolism, bicarbonate use, CO_2_ concentrating mechanisms, organic acids

## Abstract

Acclimation to variable CO_2_ was studied in floating leaves of the freshwater monocot *Ottelia cordata* grown in either low or high CO_2_. The most striking anatomical variations responding to high CO_2_ included the enlarged upper epidermal cells and the decreased area of epidermal chloroplasts. Stomata that distributed on the upper surface, and the stomatic chamber area, showed no significant response to high CO_2_. pH-drift experiments indicated that floating leaves of *O. cordata* were able to use bicarbonate regardless of CO_2_ concentrations. Photosynthetic enzyme activities and patterns of organic acids fluctuation confirmed that floating leaves of *O. cordata* can operate CAM only at low CO_2_, and perform C_4_-like metabolism at both high and low CO_2_. Overall, the present results imply that the floating leaves of *O. cordata* does not just rely on the atmospheric CO_2_ for its inorganic carbon, but is also dependent on CO_2_ and bicarbonate in the water. By showing these effects of CO_2_ variation, we highlight the need for further experimental studies on the regulatory mechanisms in *O. cordata* floating leaves, that prevent futile cycling among the three CO_2_ concentrating mechanisms (bicarbonate use, C_4_, and CAM metabolism) and the strategy for exploiting atmospheric CO_2_, as well as studies on the detailed biochemical pathway for C_4_ and CAM metabolism in this species.

## Introduction

Since preindustrial times, the atmospheric CO_2_ concentration has increased from ~280 ppm to currently over 400 ppm and is predicted to reach 800 ppm by the year 2100 (IPCC, 2014). The increasing concentration of atmospheric CO_2_ might influence plant traits, such as growth, photosynthesis, as well as the morphology and anatomy of leaves (Long et al., 2004). The responses of terrestrial plants to increasing atmospheric CO_2_ have been studied extensively (e.g. Long et al., 2004; Ainsworth and Long, 2005), however, studies on aquatic plants are sparse and mainly focus on marine species (Johnson et al., 2013; Koch et al., 2013). The effects of increasing atmospheric CO_2_ on freshwater macrophytes appear to be more intricate and might be species- and water body- specific (Hussner et al., 2019). Increasing the atmospheric CO_2_ will increase the availability of dissolved inorganic carbon (DIC) and decrease the pH in some aquatic ecosystems; this will also increase the proportion of CO_2_ and HCO_3_^-^ within the pool of DIC, within a given pH range (Pedersen et al., 2013). Nevertheless, in some cases such as the productive lakes, the aquatic ecosystems are pulled out of the air-equilibrium either by rapid photosynthesis that decrease CO_2_ concentrations or rapid decomposition of litter in the sediment that increase CO_2_ concentrations in the water. This can generate diel changes in CO_2_ concentrations of over 100-fold as well as the seasonal changes (Maberly, 1996; Schippers et al., 2004). Generally, photosynthesis in submerged aquatic plants is more sensitive and responsive to increasing CO_2_ than HCO_3_^-^ (Maberly and Madsen, 1998).

As to photosynthesis in submerged aquatic plants, CO_2_ is the preferred form of carbon and a number of species could only use CO_2_. However, about 50% of tested species have evolved strategies to use HCO_3_^-^ to handle CO_2_ limitation (Iversen et al., 2019). The problem of limited inorganic carbon supply is caused by several factors (Maberly and Gontero, 2017): (1) the CO_2_ diffusion rate in water is ~10 000 times lower than in air, which hinder the CO_2_ transport into freshwater plants through the boundary layer; (2) when the requirement for inorganic carbon by photosynthesis exceeds the supply from the environment, especially in productive systems, the CO_2_ can be depleted and its concentration close to zero. Thus, in addition to HCO_3_^-^ use, freshwater plants have evolved a diversity of CO_2_ concentrating mechanisms (CCMs) to increase further the efficiency of carbon utilization. Although much less widespread than HCO_3_^-^ use, some freshwater macrophytes possess a type of C_4_ (Bowes, 2011) or crassulacean acid metabolism (CAM)-like metabolism (Keeley, 1998). Both C_4_ and CAM implement primary carbon fixation *via* the enzyme phosphoenolpyruvate carboxylase (PEPC), that is active during either day for C_4_ or during night for CAM. Unlike terrestrial C4 plants, in aquatic species, C_4_ metabolism can be induced under the limitation of inorganic carbon, or be constitutive independent of the CO_2_ concentration (Maberly and Gontero, 2017). The frequency of aquatic species with CAM is ~9% (Maberly and Gontero, 2018), and the utilization of CO_2_ can occur during daytime, allowing the freshwater CAM plants to assimilate CO_2_ continuously (Keeley, 1998; Madsen et al., 2002), which differs from the terrestrial CAM plants. CAM metabolism is a plastic process in freshwater plants (Bowes and Salvucci, 1989), and CO_2_ concentration can affect its activity (Klavsen and Maberly, 2010; Zhang et al., 2014). Except the amelioration strategies described above, exploitation strategies are another diversity of strategies to minimize the carbon constraint in aquatic plants that involve morphological and anatomical features to give access to higher availability of free CO_2_ from the sediment or the atmosphere (Klavsen et al., 2011; Maberly and Gontero, 2018). Floating and aerial leaves of some amphibious species are the examples with exploitation strategies that could exploit more CO_2_ from the atmosphere (Sand-Jensen et al., 1992; Sand-Jensen and Frost-Christensen, 1998). The compilation of CCMs in aquatic macrophytes showed that less than 25% species with an ability to use HCO_3_^-^ have alternative strategies, which mainly comprise C_4_ and access to atmospheric CO_2_; nevertheless, CAM and C_4_ do not usually coexist in one species (Maberly and Madsen, 2002; Maberly and Gontero, 2017). The submerged freshwater plant, *Ottelia alismoides*, is hitherto the only known species to operate three CCMs (HCO_3_^-^ use, C_4_ and CAM metabolism) in the same tissue (Zhang et al., 2014; Shao et al., 2017).

In addition to the responses of CCMs to variable CO_2_ availability, the size and anatomical structure of plant leaves are also influenced by CO_2_ concentration (Radoglou and Jarvis, 1990). Enhanced CO_2_ generally increases the size of terrestrial plants (Pritchard et al., 1999). In *Brassica juncea*, high CO_2_ increased the thickness of the upper and lower epidermis, as well as the mesophyll cells of leaves (Uprety et al., 2001). While, in sorghum, the increase in growth CO_2_ resulted in a marked decrease in the thickness of the bundle sheath (Watling et al., 2000). In *O. alismoides*, high CO_2_ increased the thicknesses of the epidermal layers, the air space and mesophyll cells (Han et al., 2020). To our knowledge, there is little information in the literature concerning the effects of varying CO_2_ availability on leaf structure and the relationships between structure and function in aquatic plants.

*Ottelia cordata* (Wallich) Dandy, a member of the Hydrocharitaceae, is an aquatic macrophyte distributed in China, Cambodia, Myanmar and Thailand (Wang et al., 2019; Li et al., 2020). It is a perennial and heterophyllous plant with linear or lanceolate submersed leaves and ovate-cordate floating leaves. Cultivation experiments showed that during its early growth phases, *O. cordata* is totally submerged and it only grows submerged leaves in the first year; at the second year and after, it only grows floating leaves, which is the only part of the plant that comes into contact with air (He and Sun, 1990). It was shown recently that *O. cordata* floating leaf is a HCO_3_^-^ user and perhaps operate CAM (Cao et al., 2019). The aim here was to quantify the extent of plasticity of CCMs and leaf anatomy in this species, in response to varying CO_2_ availability in the water, to evaluate further possible effects of CO_2_ enrichment on this species.

## Materials and Methods

### Plant Material

Healthy *O. cordata* plants were collected randomly from a wild population at Haikou, Hainan Province, China. After collection, in order to eliminate the differences caused by environmental heterogeneity, the plants were cultured in plastic pots with aseptic soil and filled with water, and were illuminated with FSL T5/865 28 W fluorescence tubes in a growth room located in Wuhan Botanical Garden, with ambient temperature (13°C~17°C) and ~130 μmol photon m^-2^ s^-1^ (Li-Cor underwater sensor, UWQ, connected to a Li-Cor LI-1400 data logger) at the water surface, with a 14-h light (08:00–22:00)/10-h dark photoperiod. The light intensity condition was chosen as a trade-off between having sufficient light for photosynthesis to avoid the effects induced by low light levels on anatomy and photosynthetic physiology, and not so much light to avoid causing photodamage when at low CO_2_ concentrations. The water depth was maintained at ~30 cm during the acclimation. The transplanted plants were cultured for 3~4 weeks before they were used in the experiments.

### Response of *O. cordata* Floating Leaves to Different CO_2_ Concentrations

In the different CO_2_ acclimated experiments, the pots of *O. cordata* plants were put into the white plastic tanks (65 cm × 45 cm × 35 cm) containing tap water and incubated at two CO_2_ concentrations as described previously (Huang et al., 2018). In the high CO_2_ treatment (HC), the target pH of 7.0 was maintained between 6.52 and 7.06 by bubbling the medium with pure CO_2_ under the control of a microcomputer pH controller (model 6311, Jenco Instruments, USA), producing CO_2_ concentrations between 380~1603 μM with a mean of 941 μM. A low CO_2_ (LC) concentration was produced by the natural photosynthesis of the plants which depleted the inorganic carbon of the water, and increased pH from 8.09 to 8.91, with a CO_2_ concentration range of 3~28 µM and a mean of 15 µM. The acclimation of different CO_2_ with four replicate pots per treatment, lasted four weeks in the growth room. Over the whole period of acclimation, alkalinity, pH and temperature were measured to calculate the CO_2_ concentration using the equations reported in Maberly (1996). More information of the conditions in the treatments is shown in **Table 1**. Following the different CO_2_ acclimation, there were newly floating leaves emerged, which were harvested for anatomical structure observation and physiological parameters measurement.

**Table 1:** Conditions in the low and high CO_2_ treatments.

Conditions	Low CO_2_	High CO_2_
Temperature (°C)	14.7 ± 1.4 (12.6~16.9)	14.7 ± 1.4 (12.6~16.9)
pH^#^	8.41 ± 0.37 (8.09~8.91)	6.83 ± 0.17 (6.52~7.06)
Alkalinity (mequiv L^-1^)	1.61 ± 0.16 (1.48~1.83)	2.37 ± 0.14 (2.12~2.60)
CO_2_ (μM)	15 ± 10 (3~28)	941 ± 613 (380~1603)
HCO_3_^-^ (mM)	1.51 ± 0.17 (1.30~1.79)	2.36 ± 0.28 (2.12~2.60)

Mean values are given with s.d. and ranges are in parentheses. ^#^ calculated as a geometric mean.

### Leaf Anatomy Observation by Light Microscopy and Transmission Electron Microscopy

The youngest fully developed floating leaves in both CO_2_ treatments were sampled at 19:00 and were completed within half an hour. Then the leaves were sliced into 3 mm× 3 mm leaf fragments along the midrib. The segments were fixed immediately in 2.5% glutaraldehyde (pH 7.4) at 4°C overnight and then post-fixed in 1% OsO_4_ at 4°C for 2.5 h. The semithin sections and ultrathin sections of the leaf samples were obtained according to the method of Han et al. (2020). Semithin sections were stained with methylene blue and observed using a digital light microscope (Motic BA310). Quantitative characteristics of leaf structures including the area and size of the epidermal and mesophyll cells, as well as the air space and stomatic chamber, were measured using the Motic Images Plus 2.0 ML software. Ultrathin sections were examined and photographed under a TEM (HT7700, Hitachi, Japan). The area and size of chloroplasts and starch grains in the electron micrographs were measured with ImageJ software. At least 10 epidermal/mesophyll cells from both the upper and lower surfaces were measured per leaf from the digitized images.

### pH-Drift Experiments and Drift Parameters

The ability of *O. cordata floating leaves* to use HCO_3_^-^ was assessed in pH-drift experiments (Maberly and Spence, 1983). About 0.2~0.3 g fresh weight (FW) of leaves collected from different CO_2_ acclimated plants, rinsed in clean tap water and then incubated in 70-ml test bottles with 50 ml of test solution (equimolar concentrations of NaHCO_3_ and KHCO_3_ at an overall concentration of 1 mM). The bottles with plant leaves were sealed with glass stoppers and incubated in a growth room at 25°C and ~130 µmol photon m^-2^ s^-1^ PAR. After ~24 h continuous irradiance, the final pH of the medium was measured with pH electrode (model IP-600-9 Jenco Instruments, USA) connected to a microcomputer pH controller. The final alkalinity of the solution was measured by Gran titration (Zhang et al., 2014). The total Ci remaining at the end of pH-drift, which represents the dissolved inorganic carbon comprising free CO_2_, HCO_3_^-^ and CO_3_^2-^, was designated as C_T_. The concentration of C_T_, CO_2_ and HCO_3_^-^ in the solution were calculated from the alkalinity, temperature and pH (Maberly, 1996). The quotient of C_T_/alkalinity reflects the ability of *O. cordata* floating leaves to deplete inorganic carbon (Maberly and Spence, 1983).

### Photosynthetic Enzyme Activity Measurement

Floating leaves were harvested at 21:00 h (dusk) before the lights turn off and at 07:00 h (dawn) before the lights turn on and immediately frozen in liquid nitrogen for further measurement of photosynthetic enzyme activity. The activity of ribulose 1,5-bisphosphate carboxylase–oxygenase (Rubisco), phosphoenol pyruvate carboxylase (PEPC), pyruvate phosphate dikinase (PPDK) and NADP-malic enzyme (NADP-ME), were determined according to the methods described in Zhang et al. (2014), and were assessed from the rates of NADH disappearance or appearance at 340 nm and 25°C, using a microplate reader (Tecan M200 PRO, Austria).

### CAM Activity and Organic Acids Content

Fresh *O. cordata* floating leaves (0.2~0.5 g), were collected at 21:00 h (dusk) and 07:00 h (dawn) and immediately frozen in liquid nitrogen for further measurement of acidity and organic acids. The acidity was measured according to the method of Zhang et al. (2014).

The organic acids in *O. cordata* floating leaves were extracted and detected based on the methods described previously (Pedersen et al., 2011). About 0.2 g frozen leaves were ground and extracted with ice-cold 5% (v/v) perchloric acid using a mortar and pestle. The homogenized samples were centrifuged at 8,000*g* for 10 min, the supernatant was collected and the procedure was repeated once with ice-cold 5% perchloric acid. The supernatants were mixed and adjusted to pH 3.0~3.5 using saturated K_2_CO_3_ solution, and then centrifuged again at 4,000*g* for 5 min. The resulting supernatant was filtered through a 0.22-μm filter and transferred to vials for quantification with high performance liquid chromatography (HPLC). The component of organic acids in the extraction was separated in an Athena C18-WP (4.6 mm × 250 mm) column (CNW Technologies GmbH, Germany) maintained at a constant temperature of 30°C. The mobile phase consisted of 25 mM KH_2_PO_4_ (pH 2.5) with methanol at a constant flow rate of 0.8 ml min^-1^. The concentration of organic acids was detected at 220 nm with a photodiode array detector (PDA) coupled to a HPLC (Waters e2695 system, Milford, MA, USA). The quantitative determination of the organic acids was obtained by analyzing the chromatographic data using Data System based on the corresponding retention time and peak area of the different organic acids.

### Chlorophyll, Dry Weight, and Leaf Area

The content of chlorophyll *a* and *b* in *O. cordata* floating leaves were determined spectrophotometrically at 649 and 665 nm (TU-1810PC, Purkinje General, China) according to the method described in Shao et al. (2017). Dry weight (DW) was measured after the leaves were dried for 48 h at 80°C. Projected (1-sided) leaf area was calculated from digital photographs with AreaAna software (Huazhong University of Sciences and Technology, China).

### Statistical Analysis

The data were analyzed using SPSS 16.0 (SPSS Inc., Chicago, USA). The significance of CO_2_ treatment and sample time were determined with two-way ANOVA. The independent sample t-tests were used for the comparisons on the effects of CO_2_ concentrations on the morphological and anatomical characteristics as well as the pH-drift traits. Pearson correlation was used to test the correlation between different enzyme activities and the significance level was set at 5%.

## Results

### Chlorophyll, FW/DW, and Leaf Area

High CO_2_ did not have a statistically significant effect on the chlorophyll content, FW/DW and leaf area in *O. cordata* floating leaves, when compared with that of low CO_2_ (p>0.05; **Table 2**).

**Table 2:** Effects of low and high CO_2_ concentrations on the morphological and anatomical characteristics of the *O. cordata* floating leaves.

**Character**	**Low CO_2_**	**High CO_2_**
*Whole leaf characteristics*		
Chlorophyll a (mg/g FW)	1.24 (0.34)^a^	1.02 (0.10)^a^
Chlorophyll b (mg/g FW)	0.43 (0.13)^a^	0.32 (0.05)^a^
Chlorophyll a+b (mg/g FW)	1.67 (0.41)^a^	1.34 (0.13)^a^
Chlorophyll a/b	3.09 (1.05)^a^	3.23 (0.45)^a^
FW/DW	9.79 (1.69)^a^	9.43 (1.33)^a^
Specific leaf area (1-sided cm^2^ g^–1^ FW)	51.17 (1.68)^a^	58.97 (10.70)^a^
*Cell characteristics*		
Upper epidermis cell length (μm)	28.75 (8.27)^a^	31.73 (6.82)^a^
Upper epidermis cell width (μm)	21.57 (2.89)^a^	26.60 (4.23)^b^
Length/width of upper epidermis cell	1.33 (0.34)^a^	1.22 (0.33)^a^
Lower epidermis cell length (μm)	39.08 (7.62)^a^	35.12 (6.53)^a^
Lower epidermis cell width (μm)	31.11 (4.74)^a^	29.66 (4.02)^a^
Length/width of lower epidermis cell	1.27 (0.26)^a^	1.19 (0.19)^a^
Upper epidermis cell area (μm^2^)	643.52 (184.62)^a^	774.73 (208.08)^b^
Lower epidermis cell area (μm^2^)	1136.8 (352.82)^a^	1060.19 (193.92)^a^
Upper mesophyll cell area (μm^2^)	2191.88 (3290.49)^a^	1370.04 (427.98)^a^
Lower mesophyll cell area (μm^2^)	1027.64 (338.36)^a^	981.53 (313.47)^a^
Air space area (μm^2^)	3987.07 (2239.01)^a^	4856.12 (1542.00)^a^
Stomatic chamber area (μm^2^)	10080.64 (5179.99)^a^	6441.20 (2260.55)^a^
*Chloroplast characteristics*		
*Chloroplasts in epidermal cells*		
Chloroplast major axis (μm)	7.48 (0.82)^aA^	4.87 (1.11)^bA^
Chloroplast minor axis (μm)	3.02 (0.63)^aA^	1.95 (0.26)^bA^
Major axis/minor axis of chloroplast	2.57 (0.56)^aA^	2.50 (0.42)^aA^
Area of chloroplast (μm^2^)	17.01 (3.90)^aA^	7.29 (2.26)^bA^
Area of starch (μm^2^)	0.35 (0.26)^aA^	0.11 (0.10)^bA^
Area ratio of starch to chloroplast	0.02 (0.01)^aA^	0.01 (0.01)^aA^
*Chloroplasts in mesophyll cells*		
Chloroplast major axis (μm)	6.86 (1.08)^aA^	6.81 (1.23)^aB^
Chloroplast minor axis (μm)	2.36 (0.61)^bB^	2.80 (0.97)^aB^
Major axis/minor axis of chloroplast	3.03 (0.64)^aA^	2.66 (0.90)^aA^
Area of chloroplast (μm^2^)	12.95 (5.02)^aB^	14.84 (6.31)^aB^
Area of starch (μm^2^)	0.68 (0.74)^aA^	1.05 (0.62)^aB^
Area ratio of starch to chloroplast	0.04 (0.04)^aB^	0.08 (0.04)^aB^

Mean values (n=2~4) are given with s.d. in parentheses. Significant differences between leaves treated with different CO_2_ concentration are shown based on independent sample t-tests. Data with different lower-case letters are significantly different between low and high CO_2_ treatments (p<0.05). Data with different upper-case letters are significantly different between chloroplasts in epidermal and mesophyll cells (p<0.05).

### Anatomical Structure and Ultrastructure of Chloroplast

**Figure 1:**
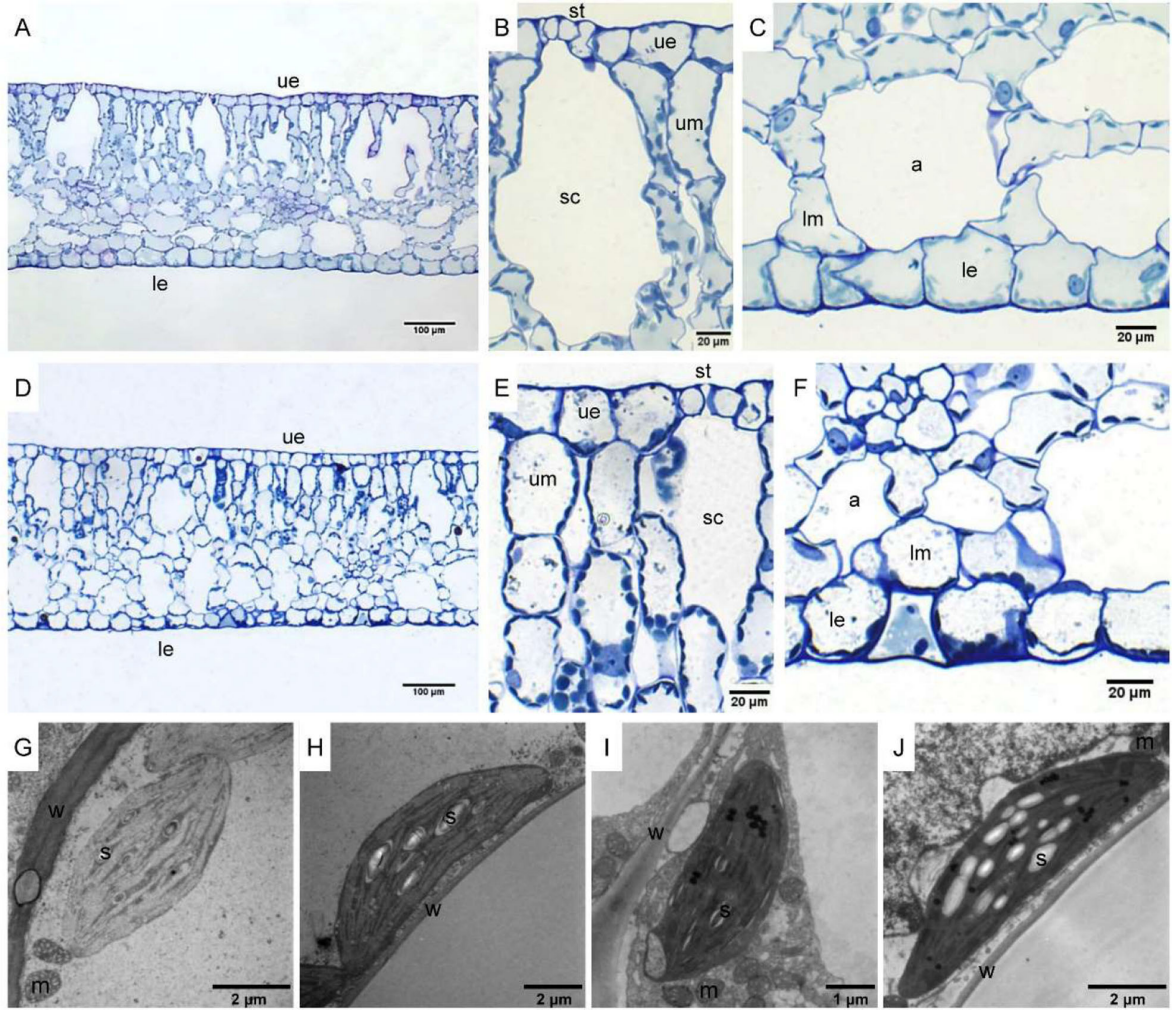
Leaf anatomy and chloroplast ultrastructure of *O. cordata* floating leaves grown at low and high CO_2_. **(A**–**C)** Anatomy of *O. cordata* floating leaf grown under low CO_2_. **(D**–**F)** Anatomy of *O. cordata* floating leaf grown under high CO_2_. **(G)** Chloroplast in epidermal cells under low CO_2_. **(H)** Chloroplast in mesophyll cells under low CO_2_. **(I)** Chloroplast in epidermal cells under high CO_2_. **(J)** chloroplast in mesophyll cells under high CO_2_. a, air space; le, lower epidermal cell; lm, lower mesophyll cell; m, mitochondria; s, starch; st, stoma; sc, stomatic chamber; ue, upper epidermal cell; um, upper mesophyll cell; w, cell wall.

The leaf anatomy of *O. cordata* floating leaves under high and low CO_2_ were shown in **Figure 1** and the basic structure of leaves did not vary with CO_2_ concentrations. The surface of *O. cordata* floating leaf was relatively flat and smooth. Transverse section observation showed that the upper and lower epidermis was single-layered rectangular cells, closely arranged (**Figures 1A–F**). The area and width of the upper epidermal cells in leaves grown under high CO_2_ were notably larger than those under low CO_2_ (p<0.05; **Table 2**), however there was no significant difference in lower epidermal cells (p>0.05; **Table 2**). The length and length/width ratio of the upper and lower epidermal cells were not significantly affected by CO_2_ concentrations (p>0.05; **Table 2**). Stomata only distributed on the upper epidermis of floating leaves of *O. cordata* (**Figures 1B, E**). The mesophyll tissues were developed, and there were air spaces interspersed among the lower mesophyll cells. The differentiation between palisade tissues and spongy tissues was present (**Figures 1B, C, E, F**). There was no significant difference in the area of air spaces, stomatic chamber, upper and lower mesophyll cells between high and low CO_2_ treated leaves (p>0.05; **Table 2**).

Considering chloroplasts can differ in cells responding to the variation of environmental factors, we evaluated chloroplast shape, size and ultrastructure in both epidermal and mesophyll cells grown at high and low CO_2_. In leaves grown at low and high CO_2_, the chloroplasts in epidermal cells were spindle-shaped (**Figures 1G, I**). The epidermal chloroplasts under low CO_2_ (**Figure 1G**) had longer length for the major and minor axis, bigger size and more starch area occupied in the chloroplast, when compared with those at high CO_2_ (**Figure 1I**; p<0.05; **Table 2**). However, the ratio of major axis length and minor axis length of the chloroplast and the area ratio of starch to chloroplast were not notably influenced by CO_2_ concentrations (p>0.05; **Table 2**). For the mesophyll chloroplast, when compared with low CO_2_ condition, except the slight but significant increased minor axis length at high CO_2_ (p<0.05; **Figures 1H, J**; **Table 2**), there was no other significant difference on the ultrastructure of this type of chloroplast (p>0.05; **Table 2**). The comparison between these two types of chloroplasts showed that, at low CO_2_, the epidermal chloroplasts had significantly larger area but lower area ratio of starch to chloroplast than mesophyll chloroplasts (p<0.05; **Table 2**). When at high CO_2_, the epidermal chloroplasts had significantly smaller size and area, as well as the smaller starch area and lower area ratio of starch to chloroplast than mesophyll chloroplasts (p<0.05; **Table 2**).

### pH-Drift Characteristics

The pH-drift experiments provided clear evidence for HCO_3_^-^ use in both CO_2_ conditions in *O. cordata* floating leaves (**Table 3**). Both high and low CO_2_ treated *O. cordata* floating leaves raised pH and reduced the concentration of free CO_2_ and HCO_3_^-^ in the medium significantly from the initial values of 7.9, 23 μM and 0.99 mM. Final pH ranged from 10.33 for high CO_2_ acclimation to 10.62 for low CO_2_ acclimation. Correspondingly, the high and low CO_2_ acclimated leaves depleted CO_2_ and HCO_3_^-^ concentrations to 17 and 4 nM, 0.18 and 0.08 mM, respectively. The [CO_2_], [HCO_3_^-^], and [C_T_] remaining at the end of pH-drift were significantly higher at high CO_2_ condition as compared with low CO_2_ acclimation (p<0.05). The quotient of C_T_/Alk, used to estimate the carbon uptake ability, was significantly lower in low CO_2_ acclimated leaves (p<0.05), indicating that the ability of using HCO_3_^-^ was more efficient under low CO_2_ condition.

**Table 3:** Final pH, alkalinity, and carbon concentrations at the end of pH-drift experiments.

Treatments	Final pH^#^	Alk	C_T_(mM)	CO_2_(μM)	HCO_3_^-^(mM)	C_T_/Alk
High CO_2_	10.33(0.12)^a^	1.04(0.11)^a^	0.49(0.05)^a^	0.017(0.008)^a^	0.18(0.04)^a^	0.48(0.05)^a^
Low CO_2_	10.62(0.16)^b^	1.06(0.12)^a^	0.34(0.10)^b^	0.004(0.002)^b^	0.08(0.04)^b^	0.32(0.09)^b^

Mean values (n=3~4) are given with s.d. in parentheses. ^#^calculated as a geometric mean. Alk means alkalinity. Data with different lower-case letters within one column are significantly different between high and low CO_2_ acclimated O. cordata floating leaves (p<0.05).

### Key Photosynthetic Enzymes Activity

**Figure 2:**
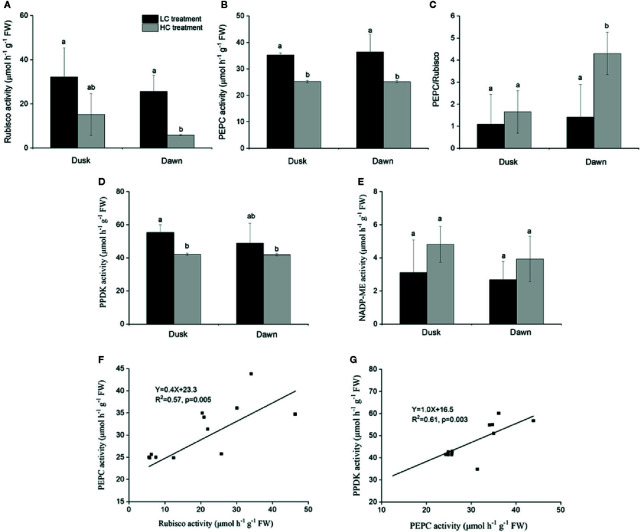
Influence of CO_2_ concentrations on activities of enzymes from *O. cordata* floating leaves collected at dusk (21:00 h, before the lights turn off) and dawn (07:00 h, before the lights turn on), as well as the correlations between activity of Rubisco, pyruvate phosphate dikinase (PPDK) and activity of phosphoenolpyruvate carboxylase (PEPC) at dusk and dawn, grown at low and high concentrations of CO_2_. **(A)** Rubisco activity. **(B)** PEPC activity. **(C)** Ratio of PEPC to Rubisco activity. **(D)** PPDK activity. **(E)** NADP-malic enzyme (NADP-ME) activity. **(F)** Correlation between activity of Rubisco and activity of PEPC. **(G)** Correlation between activity of PPDK and activity of PEPC. In (A~E), the mean values (n=3~4) with their s.d. are shown. Different letters show significant differences (*p*<0.05, *post hoc* Duncan’s test) among treatments. In (F~G), each point represents the activity measured from an individual sample from high CO_2_ and low CO_2_ acclimated plants. Pearson correlation was used to test the correlation between activity of PEPC and Rubisco, PPDK and PEPC.

Rubisco activity was 4.4-fold higher in low CO_2_ compared to high CO_2_ acclimated *O. cordata* floating leaves at dawn (p<0.05; **Table 4**), in contrast at dusk the difference was not statistically significant (p>0.05; **Figure 2A**). PEPC activity was 1.5-fold higher in low CO_2_ compared to high CO_2_ leaves (p<0.05; **Table 4**), at both dusk and dawn (**Figure 2B**). In high CO_2_ acclimated *O. cordata* floating leaves, the PEPC : Rubisco ratio was 1.7 at the end of day and increased to 4.3 by the end of night. For low CO_2_ conditions, the ratio was 1.1 at the end of day and increased slightly to 1.5 by the end of night (**Figure 2C**, **Table 4**). PPDK showed a similar pattern to PEPC (**Figure 2D**). The CO_2_ concentration triggered a 1.3-fold increase in *O. cordata* floating leaves at low compared to high CO_2_ at dusk (p<0.05), but had a slightly and not significantly promotion at dawn (p>0.05). The activity of the decarboxylating enzyme NADP-ME did not vary with the CO_2_ concentrations (**Figure 2E**). There was a significant correlation between activity of PEPC *vs.* Rubisco and PPDK *vs.* PEPC (p<0.05; **Figures 2F, G**). Activity of NADP-ME did not correlate with changes in activity of PEPC (data not shown).

**Table 4:** Two-way ANOVA results for physiological parameters in *O. cordata*, with CO_2_ concentration and time as factors.

Variables	Source					
	CO_2_		Time		CO_2_ × Time	
	F	p-value	F	p-value	F	p-value
Rubisco	12.953	**0.007**	2.446	0.156	0.071	0.796
PEPC	30.982	**0.001**	0.084	0.779	0.097	0.763
PEPC/Rubisco	25.209	**0.001**	10.504	**0.012**	6.923	**0.030**
PPDK	7.250	**0.027**	0.827	0.390	0.714	0.423
NADP-ME	2.013	0.194	0.395	0.547	0.046	0.836
Acidity	8.392	**0.016**	0.042	0.842	1.713	0.220
Malic acid	13.847	**0.006**	3.114	0.116	12.459	**0.008**
Citric acid	0.690	0.430	0.006	0.941	1.237	0.298
Aspartic acid	0.011	0.918	1.598	0.242	0.422	0.534
*trans*-Aconitic acid	0.500	0.500	0.151	0.708	0.348	0.572
Fumaric acid	141.241	**0.000**	4.736	0.061	4.972	0.056

Significant p-values (p<0.05) are shown in bold. The degrees of freedom =1 in all cases.

### Daily Oscillations of Acidity and Organic Acids

**Figure 3:**
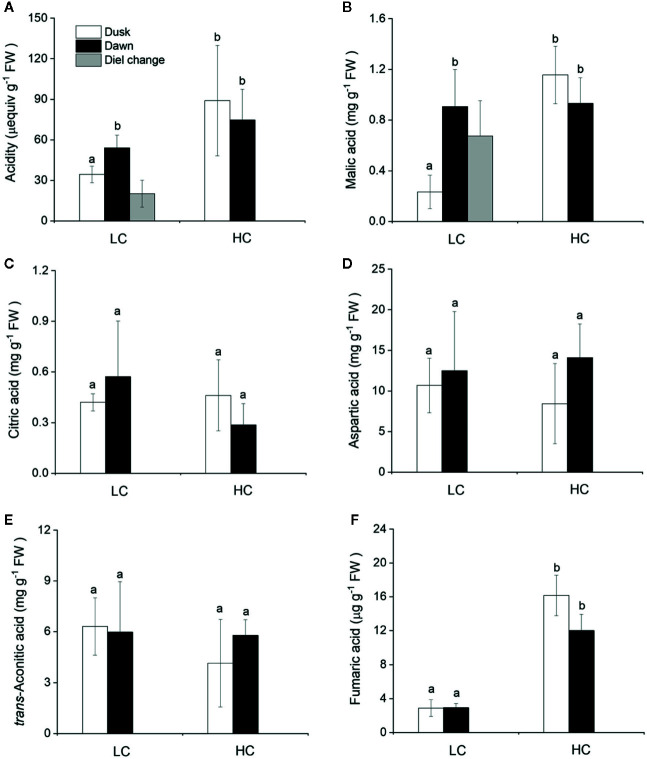
Influence of CO_2_ concentrations on acidity and concentrations of organic acids from *O. cordata* floating leaves collected at dusk (21:00 h, before the lights turn off) and dawn (07:00 h, before the lights turn on). **(A)** Acidity. **(B)** Malic acid. **(C)** Citric acid. **(D)** Aspartic acid. **(E)** *trans*-Aconitic acid. **(F)** Fumaric acid. LC means low CO_2_; HC means high CO_2_. The mean values (n=3) with their s.d. are shown. Different letters indicate significant differences (*p*<0.05, *post hoc* Duncan’s test) among treatments.

The CAM capacity in *O. cordata* floating leaves was assessed initially by measuring the diel change in acidity. Across the high and low CO_2_ conditions, acidity levels varied between 35 and 89 μequiv g^-1^ FW at dusk and between 54 and 74 μequiv g^-1^ FW at dawn (**Figure 3A**). There was a significant difference between dusk and dawn acidity levels in *O. cordata* floating leaves at low CO_2_ of about 20 μequiv g^-1^ FW (p<0.05), in contrast there was no evidence for diel acidity variation at high CO_2_.

We also analyzed organic acids levels in floating leaves of *O.cordata* at dusk and dawn. The malic acid concentrations did not differ between the two time points at high CO_2_ (p>0.05; **Figure 3B**, **Table 4**). In contrast, leaf tissues of low CO_2_ treatment showed significant dusk/dawn oscillation in malic acid content. The concentration of malic acid at dusk was only 25% of that at dawn, showing an obvious depletion of the malate pool during day period at low CO_2_ (p<0.05; **Figure 3B**, **Table 4**). However, the accumulation of malic acid at dawn did not change with the concentrations of CO_2_ (p>0.05). Concentrations of citric acid, aspartic acid and *trans*-aconitic acid in the leaf tissue did not change between the two sampling times and CO_2_ conditions (p>0.05; **Figures 3C–E**, **Table 4**). In contrast, the concentration of fumaric acid in high CO_2_ acclimated *O. cordata* floating leaves was 5.5-fold higher at dusk and 4.1-fold higher at dawn than in low CO_2_ (p<0.05), but with no significant differences between the two sampling times at both CO_2_ treatments (p>0.05; **Figure 3F**, **Table 4**).

## Discussion

### Responses of Anatomy of *O. cordata* Floating Leaves to Variable CO_2_

Through the transverse section observation of the *O. cordata* floating leaves, some anatomical changes were observed following acclimation with different CO_2_. This is in agreement with previous reports in terrestrial or aquatic plants (Thomas and Harvey, 1983; Bray and Reid, 2002; Driscoll et al., 2006; Han et al., 2020). The most striking change occurred in *O. cordata* floating leaves responding to high CO_2_ is the increased transverse-section area and width of upper epidermal cells. A similar increase of the area of upper epidermal cell in *O. alismoides* grown under elevated CO_2_ was found (Han et al., 2020). In maize, the epidermal cell size from the paradermal view was also shown to be highly responsive to environmental CO_2_ concentration (Driscoll et al., 2006). This would suggest that the enlarged epidermal cells are formed in response to high CO_2_. This response may have come about to have consequences for photosynthesis possibly by expanding CO_2_ diffusion through the upper epidermal cell membrane. Nevertheless, in some species, such as in *Phaseolus vulgaris* L. seedlings (Radoglou and Jarvis, 1992), the epidermal cells remained constant in size irrespective of CO_2_ treatment.

Stomata were found to be distributed on the upper surface of *O. cordata* floating leaves. Generally, most submerged leaves do not possess stomata, or, where they do exist but they are nonfunctional (Sculthorpe, 1967). However, floating leaves of aquatic plants, such as the aquatic pteridophyte *Marsilea*, water lilies, and *Potamogeton octandrus* have stomata on the upper surface (Lin and Yang, 1999; Rudall and Knowles, 2013; Li et al., 2019). The stomata of terrestrial plants generally distribute more on lower epidermis than upper epidermis in order to reduce water loss. However, in aquatic plants, the distribution of stomata on the upper epidermis is obviously greater than that on the lower epidermis, since the upper surface of the floating leaf is accessible to the air. Floating leaves of aquatic plants is one of the examples with exploitation strategies to overcome the problem of carbon limitation that could exploit more constant and available CO_2_ from the atmosphere based on stomata (Maberly and Gontero, 2018). Observations of the ultrathin sections showed that the epidermal chloroplast had higher sensitivity to high CO_2_ than the mesophyll chloroplast in *O. cordata* floating leaves, which differs from the responses in *O. alismoides* (Han et al., 2020), where both epidermal and mesophyll chloroplasts showed sensitive responses to CO_2_ elevation and the size of both type of chloroplasts were increased. However, it should be noted that, in both *O. cordata* and *O. alismoides*, the area ratio of starch to chloroplast was much greater in mesophyll than in epidermal cells regardless of the CO_2_ concentrations.

### Responses of CCMs in *O. cordata* Floating Leaves to Variable CO_2_

The pH-drift experiment confirmed the results reported by Cao et al. (2019), that floating leaves of *O. cordata* are able to use HCO_3_^-^. As a general trend, amphibious heterophyllous plants, in contrast to consistently submerged rooted macrophytes, are not able to use HCO_3_^-^ for photosynthesis (Madsen and Sand-Jensen, 1991). However, clearly, *O. cordata* floating leaves exhibited substantial capacity for HCO_3_^-^ utilization, irrespective of the CO_2_ concentrations during acclimation. Moreover, the HCO_3_^-^ utilization efficiency was relatively similar as that in other truly submerged bicarbonate user, such as *O. alismoides* (Zhang et al., 2014; Shao et al., 2017). Considering the much higher concentration of free CO_2_ (380~1603 μM) in high CO_2_ acclimated conditions, the finding of relatively efficient HCO_3_^-^ extraction in *O. cordata* floating leaves grown at high CO_2_ was also surprising, because at least some aquatic plants decrease investment in the capacity for HCO_3_^-^ utilization when living in an environment with sufficient CO_2_ (Maberly and Spence, 1983; Madsen and Sand-Jensen, 1987). From a cost-benefit point of view, under high CO_2_ conditions, investment in a CO_2_-concentrating system could be expected not to be profitable since the benefit in terms of carbon gain will most likely be restricted (Maberly and Gontero, 2017). However, Madsen and Maberly (1991) reported that the HCO_3_^-^ user *Ranunculus peltatus* still benefit significantly from HCO_3_^-^ usage even at very high CO_2_ concentrations (greater than 200 μM). Overall, the patterns of inorganic carbon depletion in the pH-drift system confirm that the floating leaves of *O. cordata* are a constitutive bicarbonate user.

Based on the enzymatic activity analysis, *O. cordata* floating leaves have the carboxylating, PEP regenerating and decarboxylating enzymes that are needed for operating a C_4_ pathway. We firstly compared the activities of Rubisco, PEPC, and PPDK, there are hints of possible C_4_ metabolism exist in *O. cordata* floating leaves. Under both CO_2_ acclimation conditions, the activity of PEPC was greater than that of Rubisco and the ratio of PEPC to Rubisco was about 1.5 and 4 in low and high CO_2_ conditions, respectively. The ratio for *O. cordata* is lower than *Hydrilla verticillata*, but similar to those reported for *O. alismoides*, *O. acuminata*, and *Egeria densa*, all of which are regarded as C_4_ aquatic plants (Casati et al., 2000; Bowes, 2011; Shao et al., 2017). In contrast, the PEPC/Rubisco ratio in terrestrial C_3_ plants and aquatic plants that lack a biochemical concentrating mechanism is substantially less than 1 (Zhang et al., 2014). In addition, the activity of PPDK, that regenerates PEP to ensure the supply of substrate for PEPC, was significantly greater at low *vs.* high CO_2_ and equivalent to that of PEPC. Thus, PPDK should be able to support PEPC activity. As the potential decarboxylating enzymes, the NADP-ME activity was not significantly different at low *vs.* high CO_2._ Previous work has shown that *H. verticillata* (Magnin et al., 1997) and *E. densa* (Casati et al., 2000) belong to the NADP-ME C_4_-subtype. *O. alismoides* and *O. acuminata* are the first reports of aquatic plants that belong to the NAD-ME C_4_-subtype (Zhang et al., 2014).

C_4_ metabolism in some freshwater macrophytes is induced or up-regulated under low/limited CO_2_ conditions, such as in *H. verticillata* and *E. densa* (Casati et al., 2000; Bowes, 2011). In *Eleocharis vivipara*, the C_4_ cycle is present when its leaves are in air but absent when in water (Murphy et al., 2007). In the present study, it seems that the C_4_ cycle is not abolished in high CO_2_ acclimated *O. cordata* floating leaves, which implies that C_4_ is constitutive in *O. cordata* floating leaves, like the other known two constitutive C_4_ species in the genus *Ottelia*: *O. alismoides* and *O. acuminata* (Zhang et al., 2014; Shao et al., 2017; Han et al., 2020). C_4_ plants require structural and functional coordination to operate C_4_ efficiently. For *H. verticillata*, it performs single-cell C_4_ photosynthesis, where C_4_ production and decarboxylation occur in different parts of a cell (Bowes et al., 2002). For *O. alismoides*, a dual-cell C_4_ model was assumed, where the epidermal cells produce C_4_ product and the mesophyll cells decarboxylate the C_4_ product; however, it is also possible that both processes could occur within the mesophyll cells (Han et al., 2020). For *O. cordata*, further research is needed to establish the precise location of key photosynthetic enzymes involved in the primary CO_2_ fixation and decarboxylation, which could help to clarify the specific structural basis for C_4_ cycle, as well as to confirm the C_4_-subtype in this species.

Aquatic CAM metabolism was firstly found in the freshwater lycophyte *Isoetes howellii* (Keeley, 1981), and subsequently found in other freshwater angiosperms such as *Crassula helmsii* (Newman and Raven, 1995), *Littorella uniflora* (Robe and Griffiths, 2000), *O. alismoides* (Zhang et al., 2014) and *Deinostema violaceum* (Yin et al., 2017). In CAM plants, a high nocturnal PEPC activity allows malic acid to be produced in the dark. In *O. cordata* floating leaves, PEPC remains active at night and its nocturnal activity was very high. Under low CO_2_ condition, the PEPC : Rubisco ratio was ~2, which was consistent with nocturnal carboxylation as a consequence of CAM activity. In addition, the diel fluctuations of acidity have been considered to be an important indicator to estimate CAM activity (Keeley, 1998). In the present study, only low CO_2_ acclimated *O. cordata* floating leaves exhibited marked diurnal titratable acidity fluctuation, ~20 µequiv g^-1^ FW, which was slightly lower than that found in *O. alismoides* (up to 34 µequiv g^-1^ FW) (Zhang et al., 2014). It is also lower than *Vallisneria americana* and *V. spiralis*, which present evidence for CAM operation with diel acidity changes up to 42 and 51 µequiv g^-1^ FW, respectively (Keeley, 1998). Nevertheless, similar or lower values have been reported in a number of other putative freshwater CAM species (Webb et al., 1988; Keeley, 1998). In contrast, when grown at high CO_2_, *O. cordata* floating leaves showed a small but not statistically signiﬁcant diel change in acidity, which indicates the absence of CAM. The field experiments performed by Cao et al. (2019), where the samples of *O. cordata* floating leaves were collected from their natural stands with the environmental CO_2_ concentration ~120 μM, showed the presence of CAM with 12.5 µequiv g^-1^ FW diel acidity change. This different performance of CAM activity between *O. cordata* floating leaves grown *in situ* and in laboratory cultures, might be the consequence of complex environmental variables in the field. CAM activity in aquatic plants, could be affected by a combination of factors including CO_2_ concentration, light intensity, temperature, as well as the leaf aging (Maberly and Gontero, 2017; Huang et al., 2018). Thus, the results presented here confirmed that CAM metabolism is present but facultative in *O. cordata* floating leaves.

Generally, malate is the primary product of CAM photosynthesis and presents substantial diel fluctuations (Keeley, 1998; Igamberdiev and Eprintsev, 2016). At night, the final C_4_ product malic acid is accumulated and stored in the vacuole; during day, malate comes out of the vacuole and is decarboxylated to produce CO_2_ (Borland and Taybi, 2004). In *O. cordata* floating leaves, we detected five types of organic acids and only malic acid revealed marked diurnal fluctuations at low CO_2_, the other four types of organic acid including citric acid, aspartic acid, *trans*-aconitic acid and fumaric acid were quite stable throughout the day. The significant decrease of malic acid during day suggests that it was most likely decarboxylated to produce CO_2_ for photosynthesis in *O. cordata* floating leaves when grown at low CO_2_, as it is in most of the aquatic CAM plants (Keeley, 1998). The nocturnal accumulation level of citric acid is similar with malic acid, however, the role of citrate accumulation in CAM plants is still unclear. Lüttge (1988) reported that CAM plants would transform a part of stored malate to citrate *via* tricarboxylic acid cycle (TCA cycle) to maintain their metabolic balance. Winter and Smith (1996) reported that citrate may serve to increase the pH-buffering capacity in the vacuoles of CAM plants, which would enhance accumulation of malate. The present data showed that the ratio of nocturnal citric acid accumulation to malic acid accumulation was 33% and 67% for high and low CO_2_ acclimated *O. cordata* floating leaves, respectively. In the CAM plant *Euphorbia milii*, citrate and malate accumulated equally (Herrera, 2013); whereas, Miszalski et al. (2013) found that in the CAM tree *Clusia hilariana*, malate and citrate experienced independent fluctuations, which were related to photosynthesis and respiration, respectively. In addition, in *O. cordata* floating leaves, aspartic acid and *trans*-aconitic acid presented much higher level than malic acid, but did not respond to CO_2_ availability, regardless of dusk and dawn. During metabolism the intensity of converting reactions among different carbon-compounds can cause preferential and higher accumulation of other organic acids than malic acid (Igamberdiev and Eprintsev, 2016). Moreover, aspartic acid content might be partially contributed by its derivative -asparagine, since these two amino acids are not separated quite well on the Athena C18-WP column.

## Conclusions

We conclude that the anatomical structure and CCMs in *O. cordata* floating leaves exhibits responses to CO_2_ availability. High CO_2_ acclimation increased the size of upper epidermal cells, but decreased the area of chloroplast in epidermal cells. Under high CO_2_, *O. cordata* floating leaves are able to use HCO_3_^-^ in addition to CO_2_ as an inorganic carbon source for photosynthesis, and can perform C_4_ metabolism. When exposed to low CO_2_, it also exhibits CAM cycling characteristics. Whether this species could perform other strategies under different CO_2_ conditions, such as the uptake of CO_2_ from sediment, is not known yet. Overall, the floating leaves of *O. cordata* present flexible and efficient ability to adjust structure and CCMs for utilization of inorganic carbon, allowing this species to form dense biomass and to be successfully distributed in shallow waters (He and Sun, 1990). Further studies are needed to establish the detailed biochemical pathway for C_4_ and CAM metabolism in this species, as well as the regulatory mechanism among the three CCMs and the exploitation strategies for exploiting atmospheric CO_2,_ responding to the variable CO_2_ availability in the habitat.

## Data Availability Statement

All datasets presented in this study are included in the article/supplementary material.

## Author Contributions

WH and SH designed the experiments. WH, SH, and ZX performed the experiments and collected the data. WH, SH, and WL analyzed and prepared the manuscript. All authors contributed to the article and approved the submitted version.

## Funding

This work was supported by the Strategic Priority Research Program of the Chinese Academy of Sciences (Grant No. XDB31000000) and the National Natural Science Foundation of China (Grant No. 31970368).

## Conflict of Interest

The authors declare that the research was conducted in the absence of any commercial or financial relationships that could be construed as a potential conflict of interest.
